# Influence of chlorpyrifos exposure on UVB irradiation induced toxicity in human skin cells

**DOI:** 10.1186/s12995-023-00391-5

**Published:** 2023-10-06

**Authors:** Krzysztof Sawicki, Magdalena Matysiak-Kucharek, Marcin Kruszewski, Paulina Wojtyła-Buciora, Lucyna Kapka-Skrzypczak

**Affiliations:** 1 Department of Molecular Biology and Translational Research, Institute of Rural Health, Jaczewskiego 2, Lublin, 20-090 Poland; 2 Institute of Nuclear Chemistry and Technology, Centre for Radiobiology and Biological Dosimetry, Warsaw, Poland; 3 Department of Social Medicine and Public Health, Calisia University, Kalisz, Poland; 4 World Institute for Family Health, Calisia University, Kalisz, Poland

**Keywords:** Chlorpyrifos, UVB radiation, DNA damage, Reactive oxygen species, Skin cells

## Abstract

**Background:**

Although chlorpyrifos (CPS) has been banned in many developed countries, it still remains one of the best-selling pesticides in the world. Widespread environmental and occupational exposure to CPS pose a serious risk to human health. Another environmental factor that can adversely affect human health is ultraviolet radiation B (UVB, 280–315 nm wave length). Here we attempt determine if exposure to CPS can modify toxic effects of UVB. Such situation might be a common phenomenon in agriculture workers, where exposure to both factors takes place.

**Methods:**

Two skin cell lines; namely human immortalized keratinocytes HaCaT and BJ human fibroblasts were used in this study. Cytotoxicity was investigated using a cell membrane damage detection assay (LDH Cytotoxicity Assay), a DNA damage detection assay (Comet Assay), an apoptosis induction detection assay (Apo-ONE Homogeneous Caspase-3/7 Assay) and a cell reactive oxygen species detection assay (ROS-Glo H_2_O_2_ assay). Cytokine IL-6 production was also measured in cells using an ELISA IL-6 Assay.

**Results:**

Pre-incubation of skin cells with CPS significantly increased UVB-induced toxicity at the highest UVB doses (15 and 20 mJ/cm^2^). Also pre-exposure of BJ cells to CPS significantly increased the level of DNA damage, except for 20 mJ/cm^2^ UVB. In contrast, pre-exposure of HaCaT cells, to CPS prior to UVB radiation did not cause any significant changes. A decrease in caspase 3/7 activity was observed in HaCaT cells pre-exposed to 250 µM CPS and 5 mJ/cm^2^ UVB. Meanwhile, no statistically significant changes were observed in fibroblasts. In HaCaT cells, pre-exposure to CPS resulted in a statistically significant increase in ROS production. Also, in BJ cells, similar results were obtained except for 20 mJ/cm^2^. Interestingly, CPS seems to inhibited IL-6 production in HaCaT and BJ cells exposed to UVB (in the case of HaCaT cells for all UVB doses, while for BJ cells only at 15 and 20 mJ/cm^2^).

**Conclusions:**

In conclusion, the present study indicates that CPS may contribute to the increased UVB-induced toxicity in skin cells, which was likely due to the induction of ROS formation along with the generation of DNA damage. However, further studies are required to gain better understanding of the mechanisms involved.

## Background

Due to relatively low production costs and low environmental bioaccumulation organophosphorus pesticides (OPs) are among the most commonly used pesticides all over the world [1, 2]. This includes chlorpyrifos (O, O-diethyl-O-3,5,6-trichloro-2-pyridylphosphorothioate; CPS), which has been widely adopted worldwide since the 1950s for pest control in agriculture and horticulture, as well as in domestic use for controlling flies, mosquitoes and cockroaches [3, 4]. Despite CPS use being banned in many developed countries from the early 2000s, it is still regrettably one of the best-selling pesticides in the world due to its affordability and general availability [5, 6]. Such widespread use of CPS brings the risk of unintentional exposure from water and/or food contaminated with pesticide residue [7]. The most vulnerable group through occupational exposure are farmers [3], who absorb the pesticide mostly through the dermal route that accounts for approximately 94–96% of workplace exposure [8]. CPS exposure results in neurotoxicity due to the inhibition of acetylcholinesterase activity, whereas on cellular level it is cytotoxic mainly due to induction of oxidative stress [9, 10], as a result of mitochondrial dysfunction [11].

Another harmful environmental factor that is associated with agriculture work is sunlight, more precisely an ultraviolet radiation (UV), especially UVB (315–280 nm wave length). The skin directly absorbs UVB radiation that leads to various adverse health effects including sunburn, erythema, edema, inflammation, senescence, immuno-suppression and skin cancer [12, 13]. At the cellular level UVB radiation demonstrates high genotoxic potential, due to induction of cyclobutan pyrimidine dimers (CPDs) and pyrimidine (6 − 4) pyrimidone (6 − 4 photoproducts) [14]. In addition, in skin cells UVB radiation induces excessive ROS production, which may cause oxidative damage to DNA and/or stimulate cellular signaling pathways of the mitogen-activated protein kinases (MAPK) and phosphatidylinositol 3-kinases (PI3K)/Akt [15, 16]. Activation of protein kinases affects many cellular functions, such as induction of apoptosis and activation of the transcription factors [12, 17].

It has been shown that in skin cells UVB radiation induces expression of a large number of cytokines and chemokines of both pro- and anti-inflammatory effects. Among cytokines produced in skin cells, IL-1 and IL-6 derived from keratinocytes seem to be of a particular importance [18]. Production of these cytokines leads to a cutaneous inflammatory response manifested as recruitment of immunologically competent cells, edema and fibrosis [19].

Many studies demonstrate that UVB radiation can penetrate upper layers of the skin, namely epidermis, where numerous damage to skin cells can occur; the majority being observed in keratinocytes [16]. Keratinocytes constitute the main population of epidermal cells and play key roles in the skin inflammatory response and in producing a variety of cellular factors after being exposed to various exogenous stimuli, including pesticides and UVB radiation [20, 21]. Fibroblasts are another classic type of cells commonly used to study cytotoxicity of many environmental factors, and have been shown to be responsible for delivering nutrients to other types of skin cells and maintaining the structure of the extracellular matrix [22].

Due to the likelihood of simultaneous exposure to CPS and UVB, there is a possibility of their mutual interaction. Given the widespread use of CPS in agriculture, we assumed that exposure to this compound has rather continuous character. While UVB exposure mainly depends on local weather conditions. Therefore, to mimic environmental exposure, in this study we aimed to determine an effect of chlorpyrifos pre-exposure on UVB radiation toxicity in human keratinocytes and fibroblast cells culture.

## Methods

All chemicals, culture media and supplements were purchased from Merck KGaA (Darmstadt, Germany), unless otherwise indicated.

### Cell culture

Experiments were performed on two human skin cell lines: HaCaT immortalized keratinocytes (CLS Cell Lines Service GmbH, Eppelheim, Germany) and BJ normal fibroblasts (ATCC® CRL2522™, Manassas, USA). HaCaT cells were cultured in DMEM medium supplemented with 4500 mg/l glucose, 200 mM L-glutamine, 10 U/ml penicillin, 100 mg/ml streptomycin and 10% Fetal Bovine Serum. BJ cells were cultured in EMEM medium supplemented with 200 mM L-glutamine, 10 U/ml penicillin, 10 mg/ml streptomycin and 10% Fetal Bovine Serum. The cells were cultured according to the manufacturer’s instructions at 37ºC and 5% CO_2_. Experiments were performed between 19 and 25 passage of HaCaT cells and 8–16 passage of BJ cells.

### Exposure to CPS and/or UVB

Before experiments, the cells were usually seeded in 96-well plate (Thermo Fisher Scientific, Waltham, USA) at density 1 × 10^4^ cells per well. For Comet assay and IL-6 assay the cells were seeded in 6-well plate at density 4 × 10^5^ per well. The plates were incubated for 24 h at 37 °C and 5% CO_2_ to achieve proper cell attachment. After incubation, the culture medium was gently removed from the wells and appropriate dilutions of chlorpyrifos (CPS, O,O-diethyl O-3,5,6-trichloropyridin-2-yl phosphorothioate; CAS No 2921-88-2; purity ≥ 98.0%) in the culture medium were added instead. The cells were exposed to two CPS concentrations, namely 50 and 250 µM, where 50 µM CPS concentration mimicked human environmental exposure, while concentration 250 µM was intended to reflect acute, accidental exposure [23]. Cells were incubated with CPS for 24 h at 37 °C and 5% CO_2_. After CPS incubation supernatant was gently removed, the cells were washed with warm PBS with Ca^2+^ Mg^2+^ and subsequently irradiated with UVB radiation at doses 5, 10, 15 and 20 mJ/cm^2^ using a UVA/UVB lamp with a wavelength selector (365/312 nm) filter 220 × 48 mm 2 × 8 W (VilberLourmat, France). During UVB exposure, the cells were covered with a thin layer of PBS. Intensity of UVB radiation was measured using HD2102.2 photoradiometer equipped with UVB radiation sensor LP471 (Delta OHM, Italia). Time of exposure for the highest UVB dose (20 mJ/cm^2^) was approximately 40 s. Unless otherwise indicated, after exposure to UVB radiation fresh culture medium was added to the wells. Cells were kept in an incubator for 24 h according to the manufacturer’s instructions at 37ºC and 5% CO_2_.

### LDH cytotoxicity assay

The Pierce™ LDH Cytotoxicity Assay Kit (Thermo Fisher Scientific, Waltham, USA) was used according to the manufacturer’s instructions. Briefly, 24 h after exposure 50 µl of supernatant from each well were transferred into a new 96-well plate and 50 µl of Reaction mixture were added. After 30 min incubation at room temperature in darkness, enzymatic reaction was stopped by adding 50 µl per well of Stop solution. Finally, the 96-well plate was shaken and absorbance at 490 and 680 nm was read using Omega FLUOstar Microplate Reader (BMG LABTECH, Germany). All measurements were performed in triplicate. The results are expressed as a percent of the total LDH activity of the control sample, as recommended by the manufacturer.

### Comet assay

Twenty four hours after exposure, the cells were trypsinized, harvested and dry cell pellets were frozen at -80 °C. The dry cell pellet was next re-suspended in 150 µl of PBS without Ca^2+^ Mg^2+^ and mixed 1:1 with 2% type VII agarose in PBS without Ca^2+^ Mg^2+^. A 100 µl of this suspension were then added to slides coated with 0.5% type IA agarose and incubated for few minutes. Slides were then transferred to a glass slide chamber with lysis buffer (2.5 M NaCl, 100 mM Na_2_EDTA, 10 mM Trizma base, Triton X-100 1%, pH 10) and allowed to stand for 1 h at 4 °C in darkness. After lysis, slides were rinsed in PBS without Ca^2+^ Mg^2+^. Slides were then placed in a chamber in electrophoresis buffer (300mM NaOH, 1mM EDTA, pH 14) and left for 40 min at 4 °C without any light for unwinding. The slides were next subjected to electrophoresis for 30 min (1.35 V/cm; approx. 480 mA) at 4 °C in darkness. Then, slides were rinsed 3 times in dist. H_2_O, 3 times in Tris HCl buffer (0.4 M pH 7.5) and again 3 times in dist. H_2_O, and allowed to dry. The slides were then stained with DAPI (0.5 µg/ml) at 50 µl per slide and placed in a humidity chamber overnight. The slides were read off using an Olympus BX51 fluorescence microscope (Olympus, Japan) and Komet 6.0 program (Andor, USA). One hundred comets were interrogated from each duplicate slide.

### Caspase − 3/7 assay

The Apo-ONE Homogenous Caspase-3/7 Assay (Promega, Walldorf, Germany) was used according to the manufacturer’s instructions. In brief, 24 h after exposure 100 µl of Apo-ONE Caspase-3/7 Reagent were added to each well, and the plate was left for 2 h at room temperature. Finally the plate was shaken and fluorescence (excitation 485 nm, emission 530 nm) was read using Omega FLUOstar Microplate Reader (BMG LABTECH, Germany). All measurements were performed in triplicate. For better assay consistency black opaque 96-well plates (Thermo Fisher Scientific, Waltham, USA) were used.

### ROS H_2_O_2_ assay

To measure production of H_2_O_2_, ROS-Glo H_2_O_2_ Assay (Promega, Walldorf, Germany) was used according to the manufacturer’s instructions. For this assay a white opaque 96-well plate (Thermo Fisher Scientific, Waltham, USA) where used. After final exposure to UVB radiation, 80 µl fresh culture medium was added to each well and cells were incubated for 18 h at 37 °C and 5% CO_2_. After incubation 20 µl of H_2_O_2_ Substrate solution were added and incubated for 6 h at 37 °C and 5% CO_2_. Then 100 µl of Detection solution were added to each well and incubated for 20 min at room temperature, then the luminescence was measured using the Omega FLUOstar Microplate Reader (BMG LABTECH, Germany). All measurements were performed in triplicate.

### IL-6 ELISA assay

The Human Interleukin-6 ELISA Kit (Eiaab, China) was used according to the manufacturer’s instructions to measure IL-6 production by exposed cells. After 24 h supernatants were aspirated and frozen at -80 °C.

For measurements, 100 µl aliquots of test samples, standard curve samples and blanks were added to each 96 well plate pre-coated with specific anti-IL-6 antibody. After 2 h of incubation at 37 °C excess fluid was removed and 100 µl of Detection Reagent A were added to each well. Plates were incubated for further 1 h at 37 °C and each well was washed three times with 300 µl of Wash Buffer. Detection Reagent B was then added to each well and incubated for 1 h at 37 °C. After incubation wells were washed five times with 300 µl of Wash Buffer followed by the addition of 90 µl of Substrate Solution and left for 20 min at 37 °C. Fifty microliters of Stop solution were then added to each well and absorbance was read at a 450 nm using Omega FLUOstar Microplate Reader (BMG LABTECH, Germany). Protein concentrations were calculated using a standard curve. All measurements were performed in triplicate.

### Statistical analysis

Microsoft Excel (Microsoft, Redmond, USA) or GraphPad Prism 5 software (GraphPad Software, Inc., La Jolla, USA) were used for statistical analysis. A two-way ANOVA was used to perform multiple comparisons and the Bonferroni post hoc test was used to identify significant differences. Data are expressed as mean ± SD, n = 3 (unless otherwise indicated).

## Results

### Chlorpyrifos and UVB radiation cytotoxicity

**Fig. 1:**
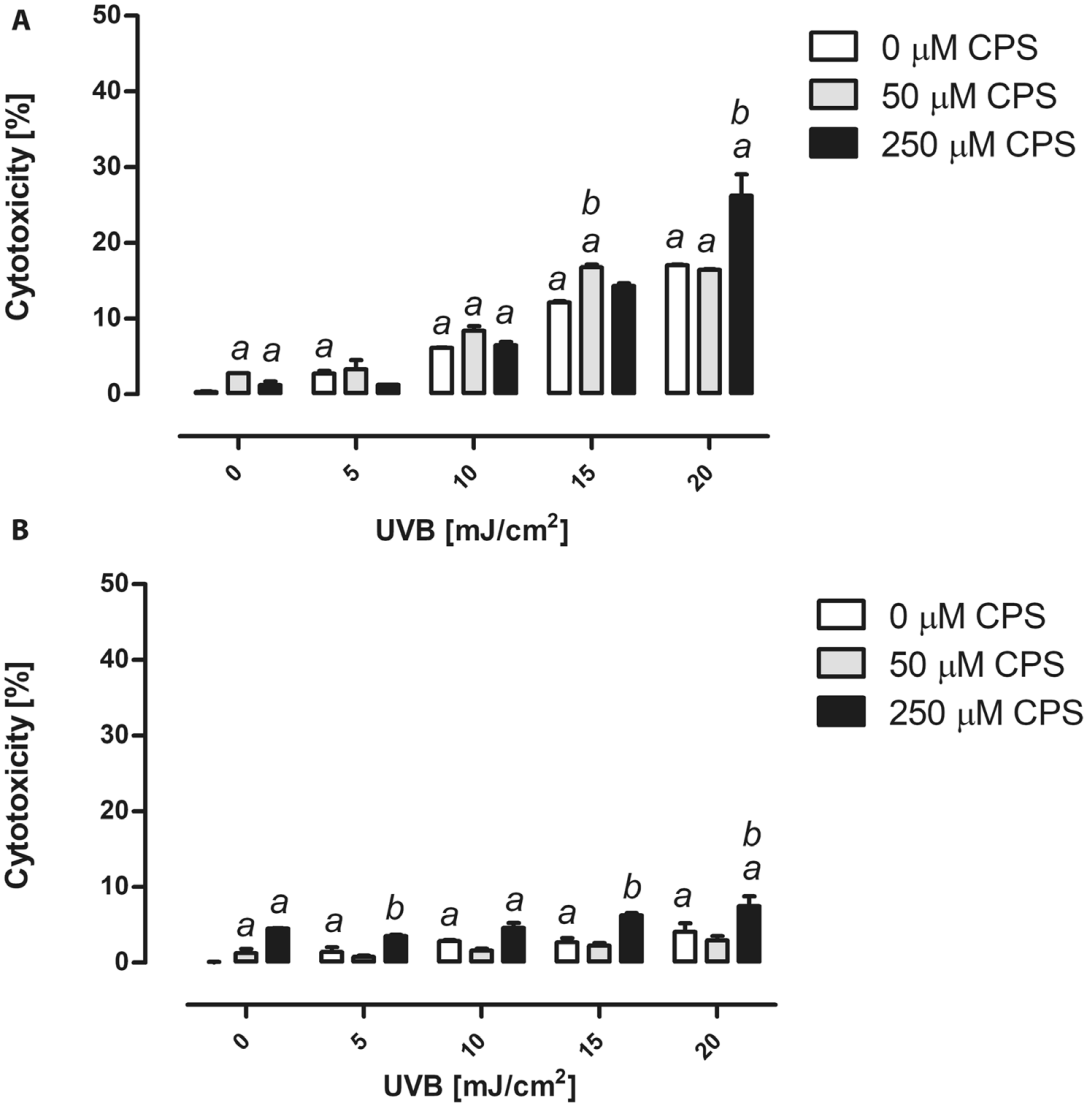
Cytotoxic effect of CPS and/or UVB irradiation on HaCaT cells **(A)** and BJ cells **(B)**. Results are expressed as a percent of the total LDH activity of the control sample. ^*a*^ denotes statistically significant difference of means from control (cells not treated with CPS nor UVB), ^*b*^ denotes statistically significant difference between CPS and UVB treated cells, as compared to UVB treatment alone. Two-way ANOVA and post-hoc comparison by Bonferroni test, *P* < 0.05. Mean ± SD, (n = 3), CPS, chlorpyrifos

Both agents were toxic to HaCaT and BJ cells, when applied alone. Fibroblasts repeatedly proved to be less susceptible to CPS- and UVB-induced toxicity than keratinocytes, with exception of the highest dose of CPS, where unexpected invulnerability of kertinocytes was observed (Fig. 1A). Exposure to the highest dose of CPS (250 µM) prior to UVB irradiation resulted in a significant increase of cytotoxicity, as compared to UVB treatment alone (Fig. 1). In keratinocytes the effect was significant for the highest doses of UVB (15 and 20 mJ/cm^2^), whereas in fibroblasts for 5, 15 and 20 mJ/cm^2^. The toxicity induced by the dose of 20 mJ/cm^2^ in CPS pre-exposed keratinocytes was more than 1.5-fold greater than when the cells were exposed to UVB alone (Fig. 1A). Similar effect was observed for fibroblasts, where cytotoxicity of UVB in CPS pre-exposed cells was 1.8-fold greater than this observed in cells exposed to UVB alone (Fig. 1B). The two-way ANOVA showed significance of interaction between CPS concentrations and UVB doses used in the experiment for HaCaT cells (*P* < 0.0001, Fig. 1A), and for BJ cells, (*P* < 0.01, Fig. 1B).

### Chlorpyrifos and UVB radiation genotoxicity

**Fig. 2:**
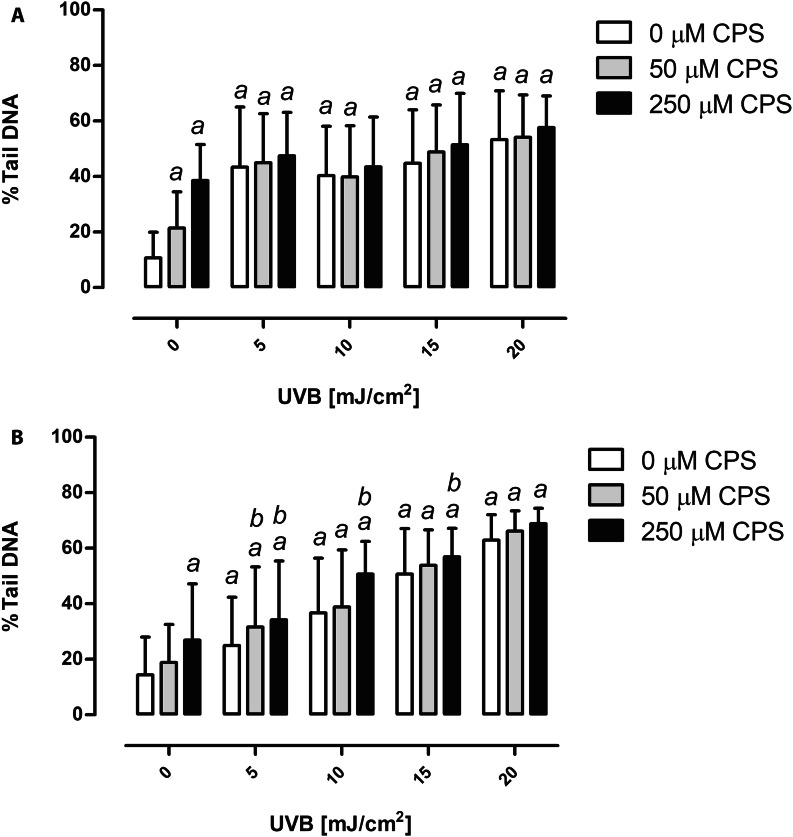
Genotoxic effect of CPS and/or UVB irradiation on HaCaT cells **(A)** and BJ cells **(B)**. ^*a*^ denotes statistically significant difference from control (cells not treated with CPS nor UVB), ^*b*^ denotes statistically significant difference between CPS and UVB treated cells and UVB treatment alone, the two-way ANOVA and post-hoc comparison by Bonferroni test, *P* < 0.05. Mean ± SD (n = 200). CPS, chlorpyrifos

Increasing CPS concentrations or UVB doses increased the level of DNA damage in skin cells when agents were applied alone. Incubation of keratinocytes with 50 or 250 µM CPS resulted in a statistically significant 2-fold or 4-fold increase in the level of DNA damage, respectively, as compared to controls (Fig. 2A). Similarly, when keratinocytes were exposed to UVB radiation, a statistically significant increase in the level of DNA damage was observed, when compared to controls. Though, a 5-fold increase in DNA damage was observed for the highest applied dose of 20 mJ/cm^2^ UVB, the observed increase was not a dose-dependent (Fig. 2A). Pre-exposure of keratinocytes to CPS did not result in any significant increase in DNA damage when compared to UVB doses alone.

Similarly, a statistically significant dose-dependent increase in the level of DNA damage was observed in fibroblasts after separate exposure of cells to the CPS or UVB radiation alone, when compared to controls (*P* < 0.05, Fig. 2B). Results also indicate that pre-exposure of BJ cells to CPS prior to UVB radiation increased the level of DNA damage, as compared to UVB alone, with exception of the highest UVB dose (20 mJ/cm^2^). The two-way ANOVA analysis revealed a significant interaction between CPS concentrations and UVB doses for HaCaT and BJ cells (*P* < 0.01 and *P* < 0.01, respectively).

### Chlorpyrifos and UVB radiation exposure modify caspase-3/7 activity

**Fig. 3:**
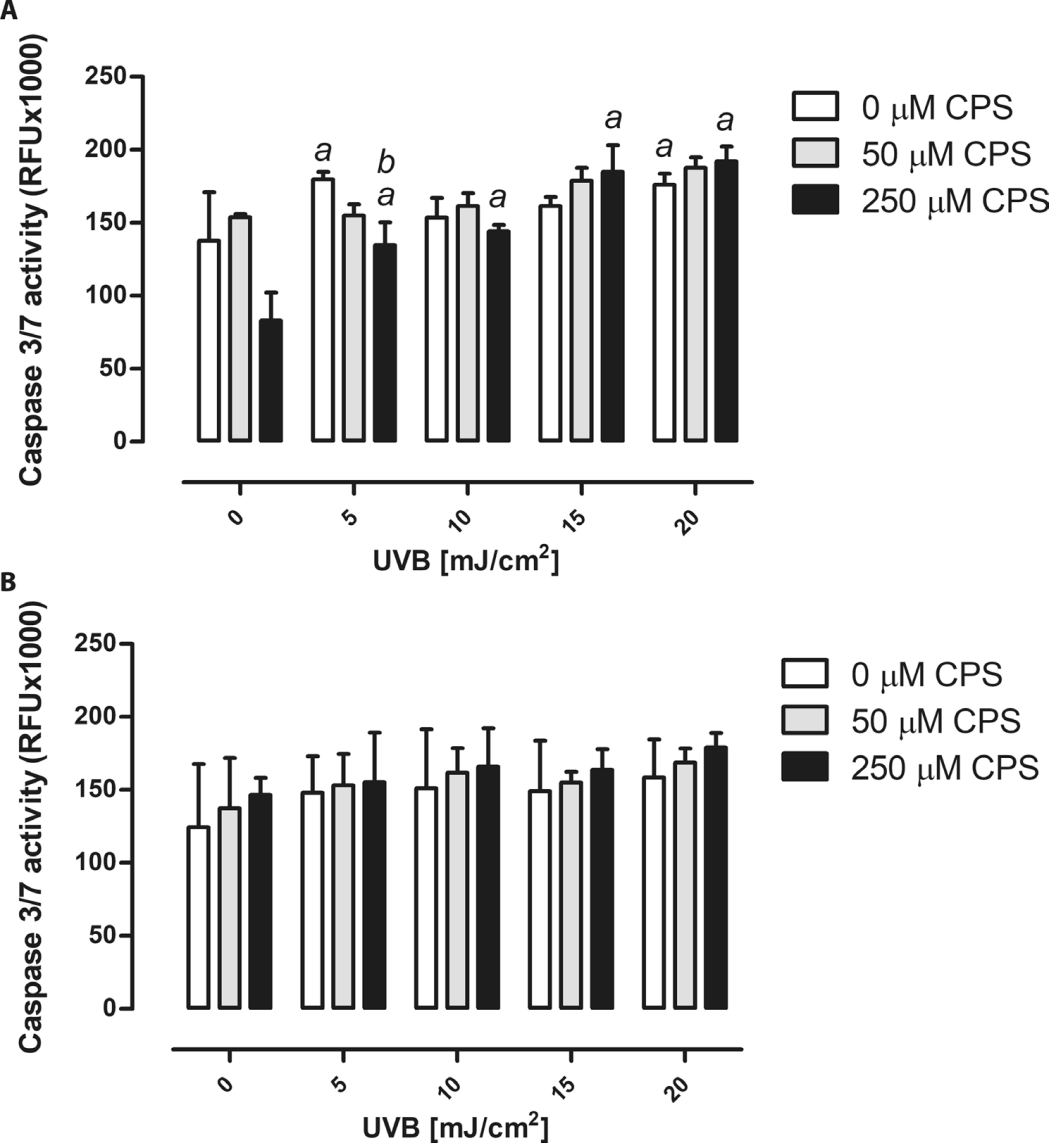
Caspase-3/7 activity measured after exposure of HaCaT cells **(A)** and BJ cells **(B)** to CPS and/or UVB radiation. ^*a*^ denotes statistically significant difference of means from control (cells not treated with CPS nor UVB), ^*b*^ denotes statistically significant difference between CPS and UVB treated cells, as compared to UVB treatment alone, the two-way ANOVA and post-hoc comparison by Bonferroni test, *P* < 0.05. Mean ± SD, (n = 3), CPS, chlorpyrifos

The study indicates that exposure of HaCaT keratinocytes to CPS decreased caspase 3/7 activity, however, these results were not statistically significant. On the other hand, seems to have exposure of HaCaT cells to UVB increased caspase 3/7 activity in a dose-dependent manner and the increase reached statistical significance at the highest UVB dose (20 mJ/cm^2^). Interestingly, the lowest UVB dose used also induced a statistically significant increase in caspase 3/7 activity, compared to control (Fig. 3A), suggesting to mode of action of UVB in keratinocytes. A decrease of caspase 3/7 activity was also observed in HaCaT cells pre-exposed to the highest dose of CPS and irradiated with the lowest dose of UVB. The increase in caspase 3/7 activity caused by the higher UVB doses apparently compensated for its reduction by CPS (Fig. 3A). The two-way ANOVA revealed that the interaction between CPS concentration and UVB doses used in the experiment was significant (P < 0.01, Fig. 3A). In contrast, no statistically significant changes in caspase 3/7 activity were found in fibroblasts, neither after treatment with CPS or UVB alone nor combined treatment (Fig. 3B).

### Chlorpyrifos and UVB radiation exposure induce production of ROS

**Fig. 4:**
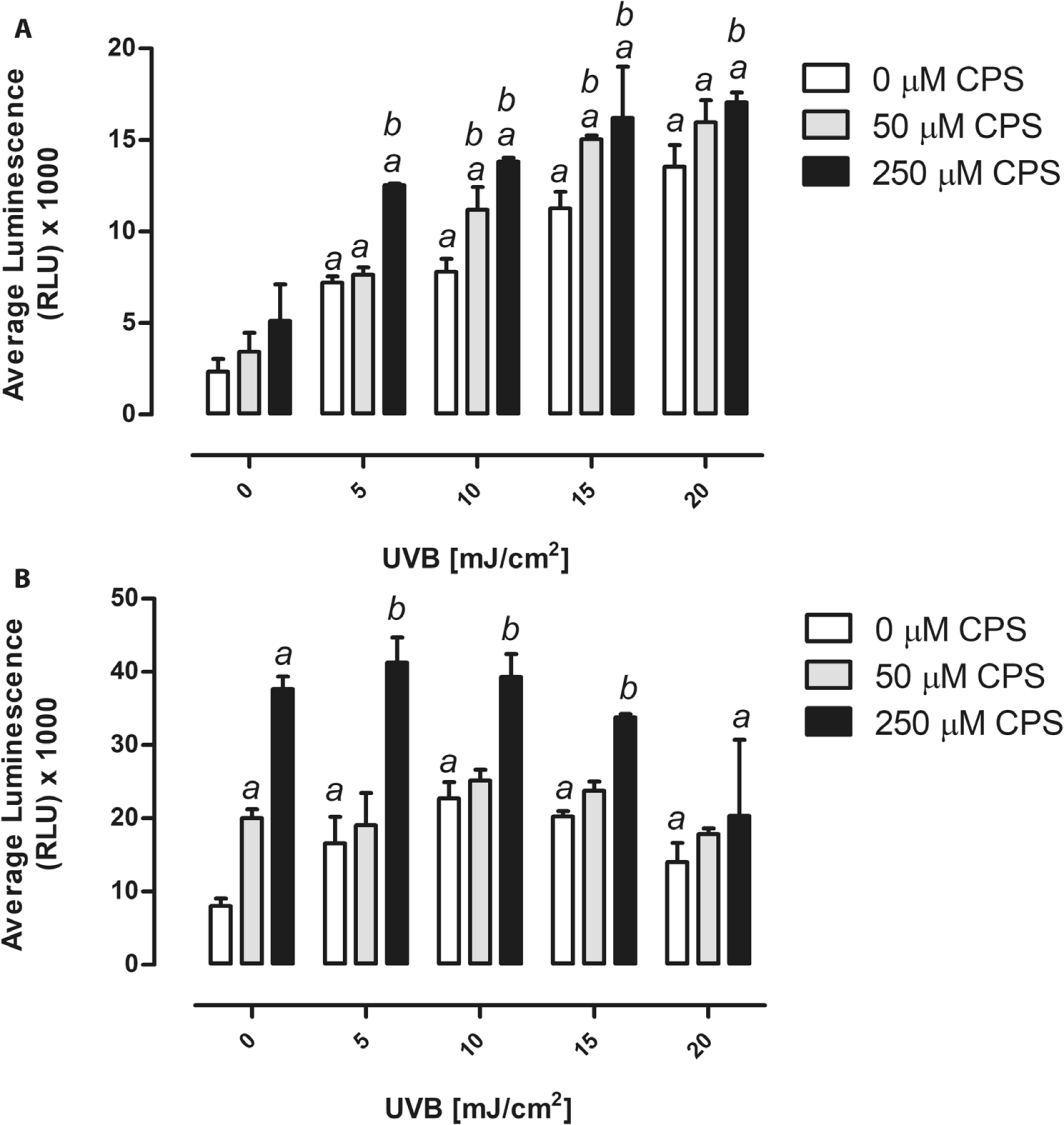
ROS production after CPS and/or UVB exposure of HaCaT cells **(A)** and BJ cells **(B)**. ^*a*^ denotes statistically significant difference from control (cells not treated with CPS nor UVB), ^*b*^ denotes statistically significant difference between CPS and UVB treated cells and UVB treatment alone. The two-way ANOVA and post-hoc comparison by Bonferroni test, *P* < 0.05. Mean ± SD (n = 3). CPS, chlorpyrifos

A statistically significant increase in ROS production was observed in keratinocytes exposed to UVB radiation alone, as compared with controls. Pre-exposure to CPS prior UVB irradiation, also resulted in a statistically significant increase in ROS production, as compared to UVB irradiation alone (Fig. 4A). Unlike to HaCaT cells, a statistically significant increase in ROS production was observed after exposure of fibroblasts to CPS alone (*P* < 0.05, Fig. 4B). Similarly, when BJ cells were exposed to increasing doses of UVB radiation, ROS production significantly increased, however the increase was not dependent on a dose (*P* < 0.05). UVB-induced ROS production significantly increased after pre-exposure of the cells to the highest dose of CPS, when compared to UVB irradiation alone, except for 20 mJ/cm^2^ UVB in fibroblasts (Fig. 4B). The two-way ANOVA showed a significant interaction between CPS concentrations and UVB doses in HaCaT keratinocytes (*P* < 0.05), and in BJ fibroblasts (*P* < 0.0001).

### Chlorpyrifos and UVB radiation exposure alter expression of IL-6

**Fig. 5:**
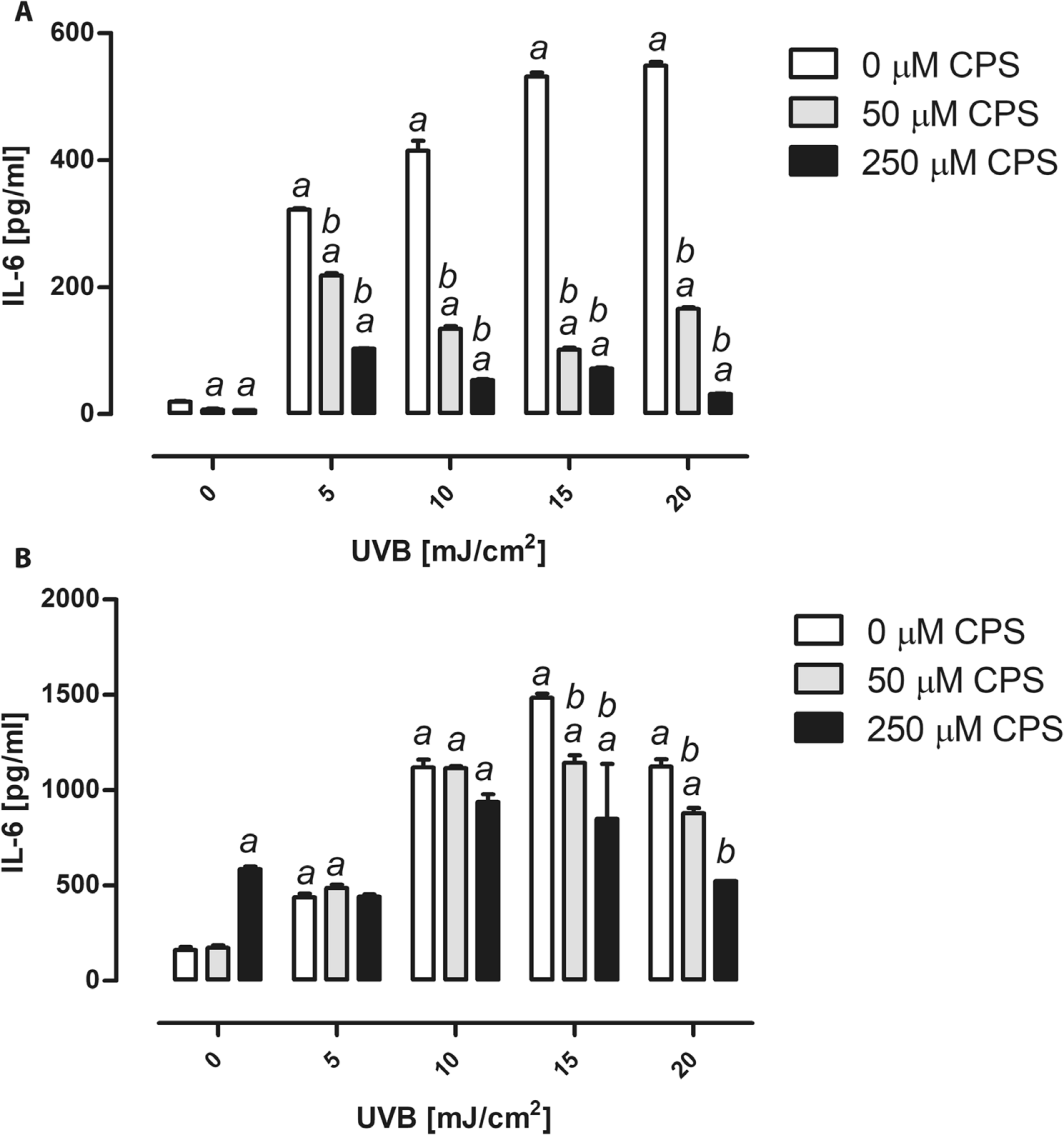
IL-6 expression after CPS and/or UVB exposure on HaCaT cells **(A)** and BJ cells **(B)**. ^*a*^ denotes statistically significant difference from control (cells not treated with CPS nor UVB), ^*b*^ denotes statistically significant difference between CPS and UVB treated cells, as compared to UVB treatment alone. Mean ± SD (n = 3). *P* < 0.05. CPS, chlorpyrifos

The treatment with CPS alone significantly reduced IL-6 production in keratinocytes (*P* < 0.05, Fig. 5A). In contrast, IL-6 production was significantly induced when keratinocytes were exposed to UVB radiation (*P* < 0.05, Fig. 5A). If compared to control, IL-6 production significantly increased also in keratinocytes pre-exposed to CPS and then irradiated with UVB, however, this increase was significantly lower when compared to UVB treatment alone (*P* < 0.05, Fig. 5A). Similar effect was observed in BJ fibroblasts. IL-6 production significantly increased after exposure to UVB and the highest dose of CPS (*P* < 0.05, Fig. 5B). Likewise in keratinocytes, IL-6 production in BJ fibroblasts was inhibited by CPS pre-exposure, but only for the highest doses of UVB (15 and 20 mJ/cm^2^), as compared with the samples exposed to UVB radiation alone (*P* < 0.05, Fig. 5B). CPS inhibit IL-6 production in HaCaT cells for all UVB doses, while in BJ cells only for 15 and 20 mJ/cm^2^). Furthermore, the two-way ANOVA showed a highly significant interaction between CPS concentrations and UVB doses used in the experiment for both keratinocytes and fibroblasts (*P* < 0.0001, Fig. 5).

## Discussion

The present study describes the effects of pre-exposure to CPS on harmful action of UVB radiation using different endpoints. Both agents induced cytotoxicity, as evaluated by LDH release from cytosol. HaCaT keratinocytes proved to be more sensitive to UVB radiation than BJ fibroblasts. Other authors have also shown a dose dependent cytotoxic potential of these two agents in human and animal cell lines [4, 16, 24]. It was suggested that mechanism of CPS-induced cytotoxicity was predominantly based on generation of oxidative stress, which resulted in excess production of oxidative damage to DNA and subsequent cell death [4]. Similarly, induction of DNA damage is likely the main cause of the UVB induced cytotoxicity, however in this case, generation of oxidative stress in accompanied by induction of bulky DNA damage, such as CPDs.

In line, both agents proved to have genotoxic potential, when used alone. CPS has been proved to be a pesticide of moderate to high-level genotoxicity in studies performed either in vitro cells or in vivo. Induction of DNA damage was found to be dose and time dependent, and in most cases as little as 24 h of exposure was sufficient to achieve genotoxic effects [24–29]. As a main mechanism responsible for CPS-induced genotoxicity, a mitochondrial malfunction and generation of oxidative stress has been proposed [11]. Increased formation of ROS due to the respiratory chain disruption modifies activity of antioxidant enzymes, such as superoxide dismutase (SOD), glutathione peroxidase (GPx) and catalase (CAT) [4, 30], further increasing CPS-induced oxidative stress, that results in induction of damage to nucleic acids and proteins, lipid peroxidation, inflammatory and immune response [11]. An alternate mechanism responsible for DNA damage due to CPS exposure involves a direct chemical damage to DNA and generation of DNA adducts. This is possible due to the presence of a phosphorus moiety in the CPS chemical structure, which acts as a substrate for nucleophilic attack [31].

Mitochondrial malfunction and generation of ROS might be also a cause of genotoxicity of UVB. Though, the main UV wavelength which excites chromophore and produces ROS is UVA, while UVB is thought to produce mainly bulky DNA damage, such as CPDs and (6 − 4)photoproduct [14, 32], there are evidences that this might be not a rule in skin cells. Lakatos et al. [33] observed UVB-dependent ROS generation in HaCaT cells due to the loss of mitochondrial membrane potential. Heck et al. [34] reported ROS generation in UVB-irradiated skin keratinocytes in vitro, due to malfunction of catalase. In line, ROS generation by UVB was observed also in cultured human skin dermal fibroblasts [35]. Since UVB-dependent inductions of bulky adducts and oxidative stress seems to be independent phenomena, it is plausible to assume that both might participate in DNA damage induction by UVB. Indeed, Khalil and Shebaby [16] observed generation of DNA strand breaks immediately after UVB irradiation, likely as an effect of ROS induced damage, and 24 h post-irradiation, likely as an effect of repair of bulky adducts. Since in our experimental design UVB induced DNA damage was assessed 24 h after exposure, it is probably a sum of residual damage induced by UVB-dependent ROS generation and repair of UVB-induced CPDs.

Further confirmation of the role of ROS in CPS- and UVB-induced toxicity comes from existing literature. Catechin and quercetin protected against CPS-induced lipid peroxidation in male rats [36], in line with vitamin C, vitamin E or their combination that were also beneficial in CPS-induced toxicity (see [37] for review). Antioxidants are also active against UVB-induced toxicity, e.g. antioxidants has been shown to ameliorate UVB-induced oxidative damage to DNA of mouse keratinocytes in culture [38] or prevent UVB-induced apoptosis in HaCaT cells [39].

Our results showed that pre-incubation of skin cells with CPS, led to the significant increase of UVB-induced cytotoxicity. Though the exact mechanism of this phenomenon must be elucidated in details, existing literature allows for some speculations. It is well documented that CPS generates oxidative stress in treated cells [11, 40, 41]. Generation of oxidative stress likely depletes concentration of low molecular weight antioxidants, such as reduced glutathione, which are the first line of defense against ROS [42]. Indeed, depletion of cellular glutathione content was observed in neuronal cells treated with CPS in vitro [43], and in hepatic cytosol [40] or red blood cells of CPS exposed rats [44]. CPS-induced glutathione depletion, should diminish cellular antioxidant defense against subsequent ROS generating agent (in our case UVB) that would result in increased cyto- and genotoxicity. Indeed, Heck et al., [34] described increased ROS production in UVB irradiated keratinocytes, when the cells were cultured in the presence of buthionine sulfoximine, a glutathione depleting agent. Our results also confirm this mechanism, as a significant increase of ROS production in UVB irradiated and pretreated with 250 µM CPS was observed (Fig. 4). An alternate mechanism that might be responsible for increase of UVB toxicity in CPS pretreated cells is UVB induced phototoxicity of CPS. Indeed, though in our experimental design CPS was washed out before UVB treatment, its remains in cytoplasm might undergo photooxidation leading to toxic products. According to Makino et al. [45], CPS has two absorption maxima (229 and 290 nm), thus considering that UVB has (280–315 nm wave length), there is very plausible possibility of absorption of UVB by CPS and photodynamic process on residual CPS. Whereas this issue was not planned to be tested during designing of this study, some information can be deducted from published data. A biologically active form of CPS is CPS-oxon (CPO), rising by desulfuration of CPS in cytochrome P-450 [46]. CPO, in turn, is rapidly hydrolyzed by esterases A and B to diethylphosphate (DEP) and 3,5,6-trichloro-2-pyridinol (TCP) [47]. The same product is formed also during photolysis of CPS by light. Research on photo-degradation of CPS by either sunlight or UVC indicates that CPO and TCP are the main products of this phenomenon [48–51]. Unfortunately no data is available for UVB-induced photooxidation. Considering the toxic properties of CPS metabolites (CPO and TCP), studies indicate that TCP is as potent an inducer of ROS the same as CPS [52, 53]. Whereas CPO, has less potential in this aspect [54]. Thus, assuming that UVB induced photooxidation products of CPS are similar to those rising during decomposition of CPS by sunlight or UVC, it is plausible to suppose that toxicity of CPS in UVB irradiated cells is a sum of toxicities of CPS metabolites and its photooxidation products, with prevalence of CPS metabolites toxicity.

Our next outcome, namely caspase 3/7 activation, gave unclear response. Exposure to the highest dose of CPS (250 µM) caused a decrease of caspase 3/7 activity in HaCaT cells, while exposure to UVB alone caused its increase. As might be expected, preincubation of HaCaT cells with CPS diminish UVB-induced caspase 3/7 activation, but only for the lowest dose of UVB. In contrast, BJ fibroblast seemed to be more resistant to caspase 3/7 activation by exposition to CPS or UVB, as no statistically significant differences were observed (Fig. 3A). Our data are in agreement with available literature in regards pro-apoptotic action of UVB. Though increase in caspase 3/7 activation observed in this study was not statistically significant, UVB doses and exposure time used in this study were much lower that doses used by others. For example Park and Jang, [55] reported significant activation of caspase-9 and − 3 after 8 h irradiation with doses 200 mJ/cm^2^ and higher, whereas doses below 200 mJ/cm^2^ did not affect caspases activation. In line, Lu et al., observed caspase-3 dependent apoptosis in HaCaT cells irradiated with 4 kJ of UVB [56]. Some studies indicate also increase of caspases 3/7 activation after CPS exposure in normal liver cells [57] or neurons [58] that is in contrast with our results were inhibition of caspases 3/7 activity was observed in keratinocytes treated with the highest dose of CPS or no induction of caspases 3/7 activity in treated skin fibroblasts. This discrepancy may be due to the different experimental design or differences in cell type, although further study are necessary to fully explain the mechanisms underlying apoptotic response in skin cells.

Analysis of IL-6 production revealed different responses of keratinocytes and fibroblast to CPS treatment. In keratinocytes CPS treatment inhibited IL-6 production, whereas in fibroblasts CPS treatment induced IL-6 production. Furthermore, and most interestingly, it has been observed that CPS is able to suppress UVB-induced IL-6 production in both cell types. Unfortunately, there are few studies describing the suppression of IL-6 by CPS. Essa et al. [59] showed that CPS had a strong inhibitory effect on IL-6 production in male rats. Significant inhibition of IL-6 production was also observed in studies conducted on splenocytes isolated from male Kunming mice [60]. More light on the explanation of the phenomenon that has occurred may be provided by a study by Singh et al. [61]. It was shown that exposure of *in utero*/juvenile mice to CPS can lead to immunosuppression. They observed that exposure to CPS resulted in increased numbers of T_reg_ cells, a reduced lymphoproliferative response to mitogens, as well as reduced production of IgM and pro-inflammatory cytokines: tumor necrosis factor α (TNF-α) and IL-6. The authors postulate that CPS had an overall inhibitory effect on the release of pro-inflammatory cytokines in lipopolysaccharide-stimulated splenocytes. Also, a study by Helali et al. [62] on isolated mouse peritoneal macrophages showed CPS dose-related reduce macrophage lysosomal activity and production of IL-1β, TNF-α. The authors suggest that the phenomenon of immunomodulation by OPs pesticides can be explained by a number of possible mechanisms including: altering cytokine gene transcription/translation, modifying surface receptors including the aryl hydrocarbon receptor (AhR), and by interfering with signaling pathways that are responsible for triggering signal in response to external agents [61, 62].

In contrast, cellular response to UVB irradiation in terms of IL-6 production is homogenous. A dose-dependent increase in IL-6 production after UVB exposure has been observed in normal human keratinocytes, HaCaT cells, mouse skin fibroblasts, immortalized human bulge stem cell line (Tel-E6E7), epidermal stem cells (ESCs) and human epithelial cells (HCE-2) [63–69]. These studies were confirmed by our results, as UVB irradiation enhanced IL-6 production in both, HaCaT and BJ cell lines.

### Limitation of the study

It should be emphasized that the presented study has some limitations. The observed differences between HaCaT and BJ cells in their vulnerability to the tested stress agents may be due to the different characteristics and origin. Though, HaCaT cells are very popular model to study homeostasis and pathology of epidermis, it should to be kept in mind that the cells were taken from the skin of 62-year old male and spontaneously immortalized in culture. In contrast, BJ cells, a normal fibroblasts cell line, were obtained from a foreskin of neonatal man. Hence, HaCaT cells likely have a significantly higher number of passages compared to BJ cells, both in vivo and in vitro, that might be associated with a significantly increased number of genetic mutations. In addition, it should be noted that these cells were cultured in media with different nutrient parameters that may also alter their response to stressors.

## Conclusions

The increased toxicity of UVB radiation was likely due to enhanced induction of ROS formation along with the generation of DNA damage in cells. This is particularly dangerous in the context of prolonged exposure to these two factors of farmers and field workers. On the other side, CPS mitigates UVB-induced production of IL-6 by skin cells that might be a positive effects in the context of further development of chronic inflammation and tumorigenesis of the skin. However, as we mentioned earlier, the presented studies have some limitations due to, among other things, the characteristics of the cell lines on which the studies were conducted. In addition, significant effects in most of the analyzed experiments were observed at the highest of the applied UVB doses (15 and 20 mJ/cm^2^). It is assumed that an hour of human exposure to sunlight corresponds to approximately 30 mJ/cm^2^ of UVB. Therefore, it is necessary to perform additional analyses based on higher doses of UVB exposure. Furthermore, to confirm or deny the conclusion presented in this study, that induced ROS generated by CPS and UVB accounts for the cytotoxicity and DNA damage it is necessary to perform some additional experiments using antioxidant agents.

In conclusion, this study indicates that CPS may contribute to increased UVB cytotoxicity in skin cells. On the other hand, further and detailed studies are needed to fully recognize the consequences of simultaneous exposure to CPS and UVB radiation, and to properly assess the real risk to agricultural workers.

## Data Availability

The datasets used and/or analyzed during the current study are available from the corresponding author on reasonable request.

## Abbreviations

AhR: aryl hydrocarbon receptor
CAT: catalase
CPDs: cyclobutan pyrimidine dimers
CPO: chlorpyrifos oxon
CPS: chlorpyrifos
DEP: diethylphosphate
GPx: glutathione peroxidase
IL: interleukin
LDH: lactate dehydrogenase
MAPK: mitogen-activated protein kinases
OPs: organophosphorus pesticides
PI3K: phosphatidylinositol 3-kinases
ROS: reactive oxygen species
SOD: superoxide dismutase
TCP: 3,5,6-trichloro-2-pyridinol
TNF-α: tumor necrosis factorα
UV: ultraviolet
